# Evaluating a handwashing with soap program in Australian remote Aboriginal communities: a pre and post intervention study design

**DOI:** 10.1186/s12889-015-2503-x

**Published:** 2015-11-27

**Authors:** Elizabeth McDonald, Teresa Cunningham, Nicola Slavin

**Affiliations:** Child Health Division, Menzies School of Health Research, Post Office Box 41096, Casuarina, NT 0811 Australia; Centre for Child Development and Education, Menzies School of Health Research, Menzies School of Health Research, Post Office Box 41096, Casuarina, NT 0811 Australia; Northern Territory Government Environmental Health Branch, Post Office Box 40596, Casuarina, NT 0811 Australia

**Keywords:** Hygiene, Handwashing, Indigenous, Australian Aboriginal, Theory of planned behaviour, Behaviour change, Social marketing, Health promotion, Evaluation

## Abstract

**Background:**

The No Germs on Me (NGoM) Social Marketing Campaign to promote handwashing with soap to reduce high rates of infection among children living in remote Australian Aboriginal communities has been ongoing since 2007. Recently three new television commercials were developed as an extension of the NGoM program. This paper reports on the mass media component of this program, trialling an evaluation design informed by the Theory of Planned Behaviour (TPB).

**Methods:**

A survey questionnaire taking an ecological approach and based on the principals and constructs of the TPB was developed. Surveys were completed in six discrete Aboriginal communities immediately before and on completion of four weeks intensive televising of the three new commercials.

**Results:**

Across the six communities access in the home to a television that worked ranged from 49 to 83 % (*n* = 415). Seventy-seven per cent (*n* = 319) of participants reported having seen one or more of the new commercials. Levels of acceptability and comprehension of the content of the commercials was high (97 % *n* = 308). Seventy-five per cent (*n* = 651) of participants reported they would buy more soap, toilet paper and facial tissues if these were not so expensive in their communities. For TPB constructs demonstrated to have good internal reliability the findings were mixed and these need to be interpreted with caution due to limitations in the study design.

**Conclusions:**

Cultural, social-economic and physical barriers in remote communities make it challenging to promote adults and children wash their hands with soap and maintain clean faces such that these behaviours become habit. Low levels of access to a television in the home illustrate the extreme level of disadvantage experienced in these communities. Highlighting that social marketing programs have the potential to increase disadvantage if expensive items such as television sets are needed to gain access to information. This trial of a theory informed evaluation design allowed for new and rich information to be obtained about community members’ beliefs, attitudes and intentions towards teaching and assisting children so safe hygiene behaviours become habit. Findings will support an evidence-based approach is taken to plan future NGoM program activities.

**Electronic supplementary material:**

The online version of this article (doi:10.1186/s12889-015-2503-x) contains supplementary material, which is available to authorized users.

## Background

In 2007, Northern Territory (NT) Government Environmental Health Officers (EHOs) developed the multi-pronged No Germs on Me (NGoM) Social Marketing Campaign to promote handwashing with soap to reduce high rates of diarrhoeal, respiratory (lung and ear) and skin infections among children living in remote NT Aboriginal communities [1]. The NGoM Program included eight (15 s) television commercials which utilised humour and a non-judgemental approach to encourage people to wash their hands after going to the toilet and changing babies’ nappies and before preparing food [2]. EHOs completed an evaluation of the social marketing component of the NGoM Program in 2008–2009 and findings included that community members’ recall of key messages was high; the knowledge of the importance of washing hands with soap at prescribed times had increased; and the number of community members who reported that they now washed their hands at prescribed times had increased [2]. However, there was concern that participants may have overestimated increases in handwashing behaviour as they desired to provide the ‘right’ response. Overestimating handwashing behaviour [3] and social desirability response bias [4, 5] are both well-recognised problems in this and other fields of research.

In 2013, limited funding became available to expand the social marketing component of the NGoM program and three additional television commercials were developed. This provided an opportunity to trial a more rigorous evaluation design and in this paper we report on evaluation key findings.

Indigenous Australians are disadvantaged across all measurable social determinants of health [6]. Australia is a resource rich country but in many remote Aboriginal communities access to functioning health hardware, for example - taps, toilets, shower or bath, and soap for handwashing is not always readily available [7, 8]. Household crowding can lead to health hardware such as toilets, taps, sinks and drainage systems not functioning or functioning poorly due to overuse [9, 10]. A lack of resources and/or poor governance of housing maintenance programs can mean that houses fall quickly into disrepair and the time taken to fix essential items such as taps and toilets can be extensive [8, 11].

In remote Australian Aboriginal communities, poor hygiene and poor living conditions are major contributors to high rates of infection among children [12]. In these communities, infections such as respiratory, skin and diarrhoeal diseases are endemic among children and chronic in nature [1]. It is common for children to have two or more infections at any one time [1]. A high burden of acute and chronic infection leads to them developing more serious conditions (for example – stunting, Bronchiectasis, Rheumatic Fever and Rheumatic Heart Disease) [13–16], disability (for example - hearing loss from Otitis Media) [1, 17, 18], poor educational and employment outcomes [18, 19], poor social outcomes (for example – unemployment and high rates of incarceration) [19] and a higher risk of chronic disease in adulthood [20].

Traditional child care practises are still in place in many remote communities. Households generally consist of an extended family group that often contain three to four generations of family members. Each member of the extended families considered ‘to share’ child care responsibilities [21]. There is limited intervention by adults in children’s activities unless danger is foreseen and children largely determine their own care needs, choosing when and what to eat, and when to wash and sleep [21]. Young children are not routinely assisted or supervised to wash their hands with soap after defaecating and before eating or to blow their nose. This leads to safe hygiene practices such as handwashing with soap at key times not becoming habitual. Child-to-child transmission is a key infection transmission route in remote communities with new born infants being colonised with streptococcus pneumonia and haeomophyllus influenza prior to six weeks of age [22–24].

Achieving behaviour change in hygiene practices in disadvantaged populations, especially among minority and disenfranchised groups, requires taking in to consideration factors such as events of history, day-to-day living practices, socio-economic circumstances and housing conditions [21, 25], as well as issues of equity and sustainability [26]. These issues can be taken into account when developing health promotion programs utilising a social marketing approach. Social marketing has become a popular approach to achieve voluntary behaviour change [27]. The success or otherwise of health promotion programs that target Aboriginal people living in remote communities and that utilise social marketing principles is unknown [2], and little or no theory informed evaluation of programs that aim to improve hygiene practices among children has occurred.

This paper reports on findings of a trial of an evaluation approach that utilised a tool that incorporated the constructs and principles of the Theory of Planned Behaviour (TPB) [28–31] and ecological theory [32] for use primarily in the remote Australian Aboriginal community setting. Evaluation objectives included to: a) identify the physical barriers that may prevent community members from easily adopting the recommended behaviours; b) assess coverage by measuring how many people have access to functioning televisions; c) measure how many people had seen the new commercials; and d) identify any change in beliefs, attitudes and behavioural intentions that might be attributed to having seen the commercials. The findings of the evaluation to assist service providers to design and deliver improved hygiene promotion programs in rural and remote Aboriginal communities.

## Methods

The evaluation was planned and implemented collaboratively between a health research institute and Territory and State Government agencies. A survey questionnaire was developed taking account of key social, economic, cultural and environmental factors previously identified as barriers to, or promoting of, handwashing with soap in remote communities [2, 21, 25] and based on the principals and constructs of the TPB [31]. Survey activities were planned to occur immediately before and on completion of the intervention. The scope of project activities was restricted due to having only 12 months to develop and deliver the intervention and complete the evaluation.

### The intervention

Three new television commercials were developed with the input of Aboriginal people living in remote communities. The television commercials were filmed in regional and remote locations and feature Aboriginal people from these areas. One of commercials targets primary school aged children and focuses on handwashing with soap prior to eating. This commercial utilises humour and the motivational factor of disgust to promote behaviour change with the goal to promote new normative behaviour [33]. This commercial reinforced the NGoM key message of preventing faeco-oral spread of disease as in the original commercials. The other two commercials focus on family members taking action to help interrupt child-to-child transmission of respiratory and other infections by teaching and assisting young children to wash their hands with soap and have clean faces (faces free of nasal discharge) before touching babies. The behaviour change motivational factor of nurture informs these commercials with the overall aim being to promote new normative behaviours [33]. All commercials are freely available on the internet [34].

Five television channels (four ‘free-to-air’ and one satellite) were contracted to intensively televise the three commercials over a four week period (11 May 2014 to 8 June 2014). Adult viewers were the target audience and the commercials were televised multiple times on a rotational basis during peak viewing periods including during Australian Football League and National Rugby League games and during programs such as Home and Away, X Factor, dancing shows, family movies, weekend specials, afternoon news and afternoon game shows. Additional ‘bonus’ screenings at non-peak viewing times were provided by all networks. The broadcast area covered was vast and included remote and rural communities across the NT and Western Australia, northern South Australia and central and far west Queensland and New South Wales. Following the period of intensive screening the commercials were withdrawn for four weeks to allow for the post intervention surveys to be completed. Televising of the commercials recommenced on the completion of data collection.

### Setting

Six remote Aboriginal communities representing three different geographical regions agreed to participate in the evaluation. Two communities are located in the Top End (TE) of the NT; two are in Central Australia (CA), and two in the Kimberley region of Western Australia (WA). All six communities are disadvantaged across all measurable social determinants of health.

Essential infrastructure and services are available in all the communities, for example, reliable water supply, sanitation and refuse management systems. Public housing of western design is provided to families that consist mostly of two to four bedrooms with one or two bathrooms with flush toilets and showers. Household crowding is common, caused by the general shortage of housing and this is exacerbated by the preference to live in extended family units and the frequent presence of relatives visiting from other communities.

Common to all communities are high rates of infection among young children and the need to improve hygiene practices to improve child health [12]. Characteristics that vary between the communities include degree of remoteness, level of access to services, population size and climatic conditions (sub-tropical, semi-arid and arid). The two TE communities are coastal communities that have populations in excess of 2000 people. Both communities are considered isolated, having limited access to a regional centre and services due to long distance and unsealed roads. Both communities are only accessible by air for approximately six months of the year due to monsoonal weather conditions, high rain fall and road closures. In the Top End the climate is characterised as generally being hot and humid. One CA community has a population of approximately 1900 and the other of approximately 500. Sealed roads are available for both communities to access the closest regional centre and services. The larger community having good access (approximately 10–20 km to travel), and the smaller community limited access owing to the need to drive approximately 400 km to access services. In CA, the climate is characterised as generally being dry and dusty and having extremes of heat and cold. Both the WA communities have populations of approximately 300. These communities are located in the far north of WA and experience not dissimilar climatic conditions to the TE of the NT. One WA community is a coastal community and is an approximately 20 min drive on a sealed road to the nearest regional centre. The other community is inland and the regional centre is located approximately 300 km away. The populations of all six communities fluctuate in size due to families travelling between communities and regional centres for cultural, social, family and sporting reasons. However, population mobility is greater in the CA and WA communities owing to having smaller distances to travel between communities and generally better road conditions.

### Participants

Convenience sampling was used to recruit survey participants. Potential participants who were meeting or transiting though public places in communities, for example, outside the community store or at the child care centre, were invited to take part in the evaluation. All Aboriginal persons aged 16 years or more who were currently residing in the community were eligible to participate. The choice of sampling method and eligibility criteria took account of a) current child care practices in communities, for example older siblings and extended family members all care for children and that infants and children move and live between households; b) the importance of not to be intrusive or to cause offence when recruiting participants; c) the limited resources and time available; and d) conducting an evaluation for purposes of evaluating a program offered by services providers and not a research study *per se*. The aim was to recruit a minimum of 80 persons from each community for both pre and post intervention survey rounds. This sample size based on the minimum number of participants advised as needed to test the internal validity of the constructs as determined by the developers of the TPB tool [31] and considered feasible by the EHOs. Participants were provided with a gift of toiletry and grooming products for completing the survey questionnaire. Purposive sampling was used to recruit two key informants from each community for informal interviews.

### Questionnaire development

**Fig. 1:**
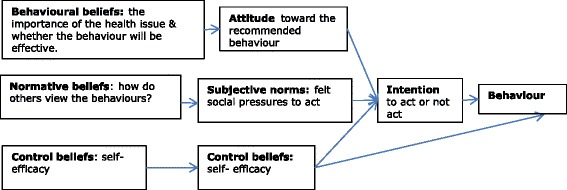
Overview of constructs of Ajzen’s Theory of Planned Behaviour (1991)

Drawing on the principles and constructs of the TPB [28–30] (Fig. 1), the questionnaire consisted of items intended to measure change concerning individuals’ beliefs, attitudes, and behavioural intentions about teaching and assisting young children to wash their hands with soap and have clean faces (faces free of nasal discharge).

**Fig. 2:**
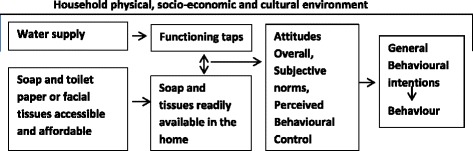
Algorithm utilising principles of Social Ecological Theory [32] and Theory of Planned Behaviour [28] showing the physical, social and cultural environmental factors important so carers’ in remote Australian Aboriginal communities are able to teach and assist children to wash their hands with soap and have clean faces

Health promotion ecological theory [32] principles informed developing questions to ascertain if the key physical, social and cultural supports identified previously as important for individuals to successfully undertake the desired behaviours were present at the time of the surveys (Fig. 2) [2, 21, 25, 32].

The pre intervention questionnaire consisted of a total of 40 items. Ten items focused on demographic and other information that might influence the way an individual responds to questions, for example – gender, age, relationship of participant to children living in the house, level of schooling, and about the level of access to a functioning health hardware and the availability or soap, toilet paper or facial tissues at the time of the survey. Toilet paper frequently used in remote communities as a cheaper and more accessible option than facial tissues for nose blowing and for cleaning nasal discharge from young children’s faces. Thirty questionnaire items were designed to measure individuals’ beliefs, attitudes, and intentions about assisting and teaching young children to wash their hands with soap and keep their faces free of nasal discharge. For example, participants were asked to score how strongly they disagreed or agreed with the following sentences:

- If you help kids to wash their hands with soap you will help stop germs spreading to babies. (Belief)
- It would make a lot of extra work for you if you were to make sure that kids always wash their hands and always have clean faces. (Attitude)
- In the future, you will make sure the kids you care for have clean faces and wash their hands with soap. (Behavioural intention)

Questions were written in such a way that some might be grouped to analyse for the four direct constructs of the TPB; Attitude Overall, Perceived Behavioural Control, Generalised Intention and Subject Norm (Figs. 1 and 2) [31]. Attitude Overall measuring if the person is in favour of doing the promoted behaviour; Perceived Behavioural Control if the person feels in control and they can undertake the behaviour; Generalised Intention if the person expects, wants or intends to comply with the promoted behaviour; and Subjective Norm measuring how much the person feels social pressure to teach and support children to wash their hands with soap and have a clean face. Internal reliability testing for the TPB constructs Attitude Overall, Perceived Behavioural Control, and Generalised Intention was satisfactory-good (Cronbach’s alpha coefficient value 0.6 or higher [35]) but poor for the construct Subjective Norm. Additional file 1: Table S1 and Additional file 2: Table S2 provide a summary of TPB questionnaire items and the internal reliability testing analysis plan and results. Questionnaire development and internal reliability and validity testing are the focus of another paper submitted for publication.

The post intervention questionnaire contained additional questions pertaining to access in the home to a functioning television; whether participants had viewed the new commercials; which commercials were seen; if the commercials were easy to understand; which commercial they liked the most; and did they learn anything new from the commercials.

Questionnaire items were written in plain English so individuals with good levels of English literacy and numeracy could complete the survey unaided and also so the questionnaire might be verbally administered to others. Likert scales were used to score TPB items and tick box responses were required for other items.

### Qualitative data

Informal interviews were conducted in each of the communities with key persons such as the manager or staff at the community store, health service staff and child care workers. The information collected focussed on what community member behaviours had generally been observed as it concerned the purchase of soap, toilet paper and facial tissues.

### Data collection

The EHOs who regularly visited and provided services in each of the communities led survey activities. They also completed the informal interviews and reported on their observations, and the key issues that arose when generally chatting with participants. The EHOs taking this role allowed for successful community engagement and also assisted later when interpreting survey findings. In all communities we endeavoured to employ one or more Aboriginal research assistants to guide evaluators in their conduct while working in their community; to provide interpreting services if required; to help recruit participants; and to administer questionnaires. EHOs and Aboriginal research assistants received instruction in the correct administration of the questionnaire.

### Data analysis

SPSS Version 22 was used to analyse the data [36]. Data was de-identified prior to any analysis and all analysis was at the population level. Analysis was conducted at community, regional and total population levels so the different geographical conditions, population size and other features, for example climatic conditions, degree of geographical isolation, may be compared. TPB constructs previously shown to have good internal reliability (Attitudes Overall, Perceived Behavioural Control and Generalised Intention) to be compared between pre and post intervention survey results using the Independent Sample t-test, and for the participants who completed both pre and post surveys the Paired Sample t-test was used [37].

Information from informal interviews and gathered when generally chatting or ‘yarning’ with participants to be examined to identify key themes to assist contextualise and explain quantitative findings.

### Ethics

Ethical approval was obtained from the Human Research Ethics Committee of the NT Department of Health and Menzies School of Health Research, the Central Australian Human Research Ethics Committee and the Western Australian Aboriginal Health Ethics Committee. Participants provided written, informed consent prior to participating in the evaluation.

## Results

Overall, a total of 865 survey questionnaires from across the six communities were completed and of this number 99 participants (11 %) participated in both pre and post surveys (Table 1). The sampling methodology used proved to be generally effective. The number and characteristics of categories of participants in each community, and across communities, reflects those who are most active in caring for children (in order - mothers, grandmothers, fathers, aunties and others). In Communities 5 and 6 difficulties were encountered recruiting the desired number of participants due to large numbers of community members being away from their communities for cultural and/or social reasons.

**Table 1:** Characteristics of survey participants' pre and post intervention from three regions (*n* 865)

Region	Gender	Age^a^			Relationship to children living in the house^b^				
	Female	>16- ≤ 25 yrs	26- ≤ 55 yrs	≥56 yrs	Mother	Father	G'mother	Auntie	Other^c^
Top End Region									
Round 1 (*n* = 163)	128 (79 %)	40 (25 %)	98 (60 %)	25 (15 %)	71 (43 %)	16 (10 %)	40 (24 %)	14 (9 %)	21 (13 %)
Round 2 (*n* = 165)	124 (75 %)	52 (32 %)	98 (59 %)	15 (9 %)	82 (49 %)	19 (12 %)	18 (11 %)	13 (8 %)	28 (17 %)
Central Australia Region									
Round 1 (*n* = 162)	120 (74 %)	35 (22 %)	105 (65 %)	20 (12 %)	48 (30 %)	20 (12 %)	49 (30 %)	13 (8 %)	31 (19 %)
Round 2 (*n* = 163)	116 (71 %)	50 (31 %)	86 (53 %)	26 (15 %)	55 (35 %)	15 (9 %)	38 (23 %)	15 (9 %)	38 (23 %)
Kimberley Region									
Round 1 (*n* = 125)	85 (68 %)	46 (37 %)	68 (54 %)	10 (8 %)	36 (29 %)	15 (12 %)	21 (17 %)	12 (10 %)	41 (32 %)
Round 2 (*n* = 87)	60 (69 %)	20 (23 %)	51 (59 %)	13 (15 %)	30 (35 %)	10 (11 %)	16 (18 %)	9 (10 %)	22 (25 %)
Total	633(73 %)	243(28 %)	506(58 %)	109(13 %)	322(37 %)	95(11 %)	182(21 %)	76(9 %)	181(21 %)

^a^Missing data: Central Australia Round 1–2 (1 %) and Round 2–1 (1 %); Kimberley Round 1–1 (1 %) and Round 2–3 (3 %);

^b^Missing data: Top End Round 1–1 (<1 %) & Round 2–5 (3 %); Central Australia Round 1–1 (<1 %) and Round 2–2 (1 %);

^c^Includes Grandfather, brother, sister, uncle, foster-mother, and other not specified

Participants reporting the presence of soap in houses by survey round and community ranged from 78 % to 100 %. Access to functioning taps, toilet paper or facial tissues to easily perform the target behaviours was similar for all communities with 80 % or more participants stating that they had these resources in their homes (Table 2).

**Table 2:** Total study population (n 865) comparison by community and survey rounds about functionality of taps and availability of soap at all sinks and toilet paper or tissues in the house where they live at the time of the survey

	Today all taps work			Today soap available at all sinks			Today toilet paper or tissues available		
	Yes	Not sure	Missing data	Yes	Not sure	Missing data	Yes	Not sure	Missing data
Community 1									
Round 1 (*n* = 80)	73 (92 %)	1(1 %)	1(1 %)	73 (92 %)	2 (2 %)	0	72 (90 %)	3 (4 %)	0
Round 2 (*n* = 82)	74 (90 %)	0	0	80 (98 %)	0	1 (1 %)	78 (95 %)	0	1 (1 %)
Community 2									
Round 1 (*n* = 83)	72 (87 %)	0	0	76 (92 %)	0	1 (1 %)	77 (93 %)	1 (1 %)	0
Round 2 (*n* = 83)	71 (86 %)	0	0	65 (78 %)	0	0	75 (91 %)	0	1 (1 %)
Community 3									
Round 1 (*n* = 81)	73 (90 %)	0	1(1 %)	67 (83 %)	0	1 (1 %)	70 (86 %)	0	0
Round 2 (*n* = 84)	81 (96 %)	0	1(1 %)	69 (82 %)	0	0	70 (83 %)	0	0
Community 4									
Round 1 (*n* = 81)	79 (97 %)	0	0	68 (84 %)	0	0	76 (94 %)	0	0
Round 2 (*n* = 79)	68 (86 %)	0	1(1 %)	68 (86 %)	0	0	72 (91 %)	0	0
Community 5									
Round 1 (*n* = 80)	75 (94 %)	0	0	70 (88 %)	0	2 (2 %)	77 (96 %)	0	0
Round 2 (*n* = 58)	53 (91 %)	0	0	50 (86 %)	0		54 (93 %)	0	0
Community 6									
Round 1 (*n* = 45)	43 (96 %)	0	0	40 (89 %)	0	0	44 (98 %)	0	0
Round 2 (*n* = 29)	29 (100 %)	0	0	29 (100 %)	0	0	29 (100 %)	0	0
TOTAL (*n* = 865)	791 (91 %)	1 (<1 %)	4 (<1 %)	755 (87 %)	2 (<1 %)	5 (<1 %)	794 (92 %)	4 (<1 %)	2 (<1 %)

Participants (*n* = 865) were approximately evenly divided about if they considered that in their community soap, toilet paper and/or tissues were too expensive. However, 75 % (*n* = 651) of participants responded to another question that they would probably buy more soap, toilet paper and/or tissues if these were not so expensive in their community.

The level of access to a television that works in the house ranged from 49 % to 100 % across all communities (Table 3). Seventy seven percent (77 %) (*n* = 319) of participants reported having seen one or more of the new NGOM commercials (Table 3).

**Table 3:** The number of participants who reported having a television that works in their home and who had seen the new commercials

	Working television in the house			New commercials seen		
	Yes	No	Missing data	Yes	No	Missing data
Top end						
Community 1 (n 82)	68 (83 %)	12 (15 %)	2 (2 %)	67 (82 %	13 (16 %)	2 (2 %)
Community 2 (n 83)	41 (49 %)	42 (51 %)	-	46 (56 %)	35 (42 %)	2 (2 %)
Central Australia						
Community 3 (n 84)	75 (89 %)	9 (11 %)	-	80 (95 %)	4 (5 %)	-
Community 4 (n 79)	61 (77 %)	16 (20 %)	2 (3 %)	53 (67 %)	23 (29 %)	3 (4 %)
Kimberley						
Community 5 (n 58)	44 (76 %)	14 (24 %)	-	45 (78 %)	13 (22 %)	-
Community 6 (n 29)	29 (100 %)	-	-	28 (97 %)	1 (3 %)	
Total	318 (77 %)	93 (22 %)	4 (1 %)	319 (77 %)	89 (22 %)	7 (2 %)

Three hundred and eight (97 %) participants reported they liked the new commercials – 29 % rated the commercial featuring the father and son as the one they liked the most; 32 % rated the commercial featuring boys playing football as their favourite; and 19 % rated the commercial featuring the girls playing ‘clap hands’ as the one they liked the most (10 % of participants could not decide which they liked the best and missing data accounted for 10 % cases). Three hundred and eleven (98 %) participants reported they understood the ‘stories’ in the commercials.

In general, for both survey rounds, all participants scored individual questionnaire items (40 items in total) towards the high end of scales if not provided a perfect score. Therefore mean scores for items were high at community, regional and total population levels pre and post intervention and any difference in mean scores for items between survey rounds were small.

The comparison of pre and post intervention survey results by community using the Paired Sample t-test for the 99 participants who completed both surveys generated mixed results - that is positive and negative changes as well as no change (Additional file 3: Table S3). The size of any change was small and in only two cases was the change statistically significant at the 5 % level of significance. This was for Communities 3 and 5 for the TPB construct Attitude Overall (Community 3 *p* < .008 and Community 5 *p* < .00).

Paired sample – t-test analysis at the regional level (Top End, Central Australia, and Kimberley) and for All Communities for the TPB constructs considered reliable revealed a statistically significant change for the construct Attitude Overall for Kimberley Region (*p* < 0.000) and for All Communities (*p* < 0.041) (Table 4).

**Table 4:** Results of individual and combined regional level Paired Sample t-test (pre and post intervention) – Theory of Planned Behaviour constructs shown to have good internal reliability

	Number of participants	Mean^a^	Standard deviation	Correlation	Significance
Attitude overall					
Top End Region					
Pre	28	6.20	1.75	−0.111	0.573
Post	28	6.54	0.60		
Central Australian Region					
Pre	45	6.60	1.12	0.212	0.163
Post	45	6.70	0.72		
Kimberley Region					
Pre	26	6.60	0.91	0.762	0.000**
Post	26	6.75	0.68		
All communities					
Pre	99	6.49	1.29	0.205	0.041**
Post	99	6.67	0.68		
Perceived behavioural control					
Top end region					
Pre	28	5.31	1.67	0.038	0.847
Post	28	4.95	1.46		
Central Australian Region					
Pre	45	6.40	0.95	−0.121	0.429
Post	45	5.89	1.87		
Kimberley Region					
Pre	26	5.42	1.88	0.324	0.106
Post	26	5.47	1.97		
All communities					
Pre	99	5.83	1.53	0.151	0.136
Post	99	5.51	1.82		
Generalised intention					
Top End Region					
Pre	28	5.15	2.26	−0.183	0.351
Post	28	5.10	2.01		
Central Australian Region					
Pre	45	6.29	1.22	−0.132	0.388
Post	45	6.06	1.89		
Kimberley Region					
Pre	26	5.80	2.01	0.324	0.106
Post	26	6.23	1.68		
All communities					
Pre	99	5.84	1.82	.030	0.765
Post	99	5.83	1.91		

^a^Construct scale score range 1 – 7 **Significance at *p* = <0.05 level

To obtain an independent population sample to enable Independent Sample t-test analysis data belonging to the 99 participants who participated in both survey rounds were removed from the total sample (*n* = 865) thus leaving responses from 766 different individuals. This analysis showed no differences of statistical significance (*p* < .05) for the communities combined and Communities 2, 3, 5 and 6. For Communities 1 and 4 statistically significant changes (*p* < .05) were observed (Table 5).

**Table 5:** Results of Independent Sample t-test – Theory of Planned Behaviour constructs shown to have satisfactory-good^a^ internal reliability

Theory of planned behaviour - construct	Pre Survey Mean^b^ (SD)	Post Survey Mean^b^ (SD)	Sig (2-tailed)***	95 % confidence interval	
				Lower	Upper
Community 1 (Top End Region)					
Attitude overall	6.0	6.6	.006	−1.02	−0.18
Cronbach alpha pre 0.84	(1.3)	(1.2)			
Cronbach alpha post 0.53					
Perceived behavioural control	4.6	5.7	.001	−1.74	−0.43
Cronbach alpha pre 0.53	(2.2)	(1.7)			
Cronbach alpha post 0.70					
Community 4 (Kimberley Region)					
Perceived behavioural control	6.2	5.3	.002	0.34	1.43
Cronbach alpha pre 0.53	(1.3)	(1.9)			
Cronbach alpha post 0.70					

^a^Cronbach alpha satisfactory at 0.60 level ^b^Construct scale score range 1–7. ***Significance at *p* = <0.05 level

For Community 1 the differences in the means were all in the positive direction but for Community 4 these were in the negative direction.

Eight interviews with Aboriginal participants (senior women in the community, health workers and shop and child care assistants) and four with non-Indigenous participants (nurses and store managers) were completed. Several common key themes emerged from the interview data and the additional information learnt from informally chatting with participants, including:

i) those caring for children ‘tell’ and ‘shout at’ children to wash their hands and clean their faces but children do not do as they are told and run away. For example, an Aboriginal participant interviewed stated:
  *Most of kids don’t like being cleaned and make a real fuss. Some parents give into the kid making a fuss and don’t make them do what they are told.*
  Another said:
  *Kids try and run away but good parents chase them*.
ii) families often do not have soap, toilet paper or facial tissues readily available in the home. An Aboriginal mother stated:
  *Families use shampoos, rinso or soap for personal hygiene. Sometimes they have nothing and try to borrow soap.*
  A health centre staff member reported community members frequently ask to be given soap and other hygiene products because they have no money to buy these items.
iii) families often do not have the money to buy soap, toilet paper or face tissues and buying other essential and non-essential items has greater priority, an Aboriginal participant interviewed reported:
  *The store is too expensive. People buy in order - food, toilet paper but they don’t think washing hands is an emergency. People think that hand soap, shampoo, deodorant, are luxury items.*
  Another Aboriginal participant stated:
  *People try to buy but if they don’t have money they buy food first.*
  In contrast a non-Aboriginal person interviewed reported:
  *…. a lot of people aren’t buying soap. I never see people buying hygiene items in the store. Heaps of Aboriginal people believe soap is not needed. Hasn’t been used in the past, ‘its high class’ and ‘the white way’. Community will use what we give them but they won’t spend their own money. They prefer to use it to buy smokes, alcohol or gamble.*

## Discussion

Evaluation findings highlight that there are still physical and socio-economic barriers present in remote communities, such as the functionality of taps and the ready availability of soap, toilet paper or facial tissues, that prevent those caring for children easily meeting children’s basic hygiene needs such as handwashing with soap and facial cleanliness. The underlying reasons for this are complex and not amenable to simple solutions. Until the current physical and socio-economic barriers to achieving hygiene behaviour change are reduced, achieving hygiene behaviour change so as to reduce the burden of infection among Aboriginal children living in remote communities will be largely ineffective [32, 38].

Recent major housing construction and renovation programs carried out in many remote Aboriginal communities have resulted in increased functionality of health hardware in houses [8]. However, as mentioned earlier in this paper, household crowding leading to high usage means health hardware requires close monitoring and repair more frequently. The proportion of participants (range 3 – 17 %, Table 2) who reported that not all taps in their house work is of concern because there is the potential that non-functional health hardware will once again reach previously high levels [7]. The frequent changing of housing policy and programs (to meet immediate political imperatives rather than need), and the variability across communities as to the resources available and the efficiency and effectiveness of housing repairs and maintenance programs, means that the lack of timely action will result in the condition of houses quickly deteriorating [10].

Survey and qualitative findings both support that easy access to soap and toilet paper or facial tissues remains poor (Table 2). This particularly the case for one community where for one survey round only 78 % of participants reported there was soap in their houses (Table 2). This low figure, and the higher numbers reported in the case of the other communities, is likely to include over reporting as this is generally the case with similar surveys when participants desire to provide the perceived socially correct answer [4]. This perception that over reporting occurred is supported by store managers who stated that the sale of soap and other hygiene products is generally poor. EHOs who often visit homes, especially to view the condition of kitchens, bathrooms and toilets, also report they generally do not see soap near sinks nor toilet paper or facial tissues in homes. In the developing country context, a satisfactory standard is that soap be *observed* to be present in 97 % of homes in a community [3, 39, 40]. Poor ready access to soap and toilet paper or facial tissues means that the items considered essential for handwashing with soap and safely maintaining children’s faces free of nasal discharge are not always available so that their use and the associated behaviour becomes a habit [3].

The high cost of soap and toilet paper and tissues in remote communities is one contributing reason for why community members do not buy these items in the quantities required to sustain safe hygiene behaviours. A number of additional factors support this finding including: a) most families in remote communities have low incomes obtained through low paid jobs or welfare benefits [41]; the overall high cost of living in remote communities acerbates socio-economic disadvantage [42]; and hygiene products in remote communities cost more than in urban and regional centres [41, 42]. In developing countries it has been noted that the availability of soap in households increases according to level of income [3].

Another reason for the lack of soap, toilet paper or facial tissues in houses is that some community members view these items, but soap especially, as non-essential purchases. It appears that when householders have sufficient money, for example when they first receive their fortnightly income payments, soap and other hygiene and grooming products are purchased. However, much of this income is spent over the first two to three days and then families have only a small amount left which must last until their next payment arrives. In this circumstance, food and other items perceived as essential or highly desirable (for example cigarettes) are purchased rather than products such as soap or toilet paper or tissues.

The level of access in the home to a television that works varied between communities, and low access (49 % *n* = 41) was recorded for Community 2 (Table 3). Overall health and social outcomes for Community 2 are worse than for the other remote communities with the underlying reasons for this being complex and not well understood. Communities 1 and 2 share many common characteristics including both are in Top End of the NT and the level of household income would be similar. However, 83 % of participants (*n* = 68) from Community 1 reported access to a television that works in the house compared to 49 % in Community 2 (Table 3). A potential reason put forward for why there was such low access to working televisions in houses in Community 2 was that damage to property such as televisions during times of community and family conflict is common. This finding highlights that relative disadvantage exists within and across remote Aboriginal communities and measuring disadvantage should not be restricted to comparing only that between broad Indigenous and non-Indigenous population groups. In the wider Australian population items such as microwave ovens and dishwashers are now used to measure relative disadvantage and television ownership is a given [43]. Thus, relatively low levels of access to a television in the home further highlights the extreme level of disadvantage experienced across remote Aboriginal communities.

The geographical reach and population coverage achieved, and the number of participants who reported seeing the commercials, is considered satisfactory given the short length of time the commercials were televised (four weeks) (Table 3). It is not surprising that in Community 2 only 58 % of participants reported having seen the commercials given the low level of access to televisions reported by participants. A key learning from this finding is that social marketing and other health promotion strategies have the potential to compound disadvantage if these rely solely on ownership or ready access to costly items such as television sets [26].

That the vast majority of participants who saw the new commercials (*n* = 308, 97 %) liked them reflects the good use of formative research and the appropriateness of the process taken to develop the content of the commercials. Positive feedback concerning the commercials was received from many quarters with only one complaint received, this from a non-Aboriginal person who lived in a regional centre and who requested that the ‘disgusting’ commercials not be screened at meal times.

A clear limitation of this evaluation was the absence of control communities, thus any change in beliefs, attitudes and behavioural intentions that might be attributed to having seen the commercials need to be interpreted with caution. The work presented here is considered exploratory in nature as the TPB constructs are generally applied to address different behaviours and in populations and contexts that differ from those in this evaluation. We perceive a further limitation was the short time over which the commercials were aired (four weeks) before the post intervention surveys were conducted. This factor likely contributed to the mixed quantitative results obtained. A further limitation in the evaluation design was not including questions that could take account of participants’ general state of mind or mood at the time of completing the survey. We consider that participants may score items about their level of self-efficacy lower when feeling physically tired, when the overall mood is low due to recent adverse personal or family experiences, or if feeling tired and frustrated from recent dealings with a mostly uncooperative child. An otherwise strong sense of self-efficacy may change when performing a behaviour that requires the co-operation of a reluctant other [44]. In general, meeting young children’s hygiene and nutrition needs is challenging but this likely applies more in the context of remote Aboriginal communities where children have a strong sense of autonomy [21].

The outcomes of this trial of a theory informed evaluation have proved to be very informative and useful for the public health practitioners who deliver hygiene promotion programs in remote Aboriginal communities. For example, generally all participants in both survey rounds provided a high score, if not a maximum score, to individual questionnaire items concerning their beliefs that handwashing with soap and facial cleanliness can interrupt child to infant spread of infection. Thus, indicating a generally good level of knowledge on this topic and that future hygiene improvement promotion that target adults in remote communities should focus less on providing education programs and more on working in the challenging areas of changing environmental factors and fostering new social norms. The evaluation findings found to be common across communities, regions and overall suggest these may generally apply to all remote Australian Aboriginal communities. Findings concerning pre and post intervention change of TPBs constructs are encouraging as to the effectiveness of the commercials and the social marketing approach taken. Other findings identify where future action and advocacy is required, including increasing the access and availability of soap; promoting the greater use of soap for handwashing and bathing children; and monitoring the level of functionality of health hardware in houses and advocating for speedy repair of non-functional items. The evaluation design, methodology used and findings are likely to prove useful for others to use in the future as a basis to plan and conduct further research on this topic in this same or similar contexts.

## Conclusion

The geographical reach and population coverage achieved, and the number of participants who reported seeing and liking the commercials, was satisfactory given the short length of time the commercials were televised. Pre and post-intervention analysis showed no changes in participants’ beliefs, attitudes and behavioural intention that might be confidently attributed to them having seen the commercials. However, taking an ecological approach and examining participants’ beliefs, attitudes, sense of self-efficacy, social norms and other elements of the TPB provided for obtaining rich information which is useful beyond reporting on the outcome of this evaluation. The findings of this evaluation will support an evidence - based approach is taken to plan and evaluate future NGoM program activities.

## Additional files

Additional file 1: Table S1. Summary of Theory of Planned Behaviour questionnaire items and internal reliability testing analysis plan. (DOCX 14 kb) Additional file 2: Table S2. Reliability tests of constructs using Cronbach’s alpha statistical test*. (DOCX 14 kb) Additional file 3: Table S3. Results of community level Paired Sample t-test (pre and post intervention) – Theory of Planned Behaviour constructs shown to have good internal reliability. (DOCX 20 kb)

## Abbreviations

CA: Central Australia
EHO: Environmental Health Officer
NGoM: No Germs on Me
NT: Northern Territory
TPB: Theory of Planned Behaviour
WA: Western Australia
