# Supplementary material for: Evaluating a handwashing with soap program in Australian remote Aboriginal communities: a pre and post intervention study design
Source: BMC Public Health. 2015 Nov 27;15:1188. doi: 10.1186/s12889-015-2503-x (PMC4662811; doi:10.1186/s12889-015-2503-x)
Supplement: Additional file 1: Table S1. — Summary of Theory of Planned Behaviour questionnaire items and internal reliability testing analysis plan. (DOCX 14 kb) [file 12889_2015_2503_MOESM1_ESM.docx]

**Additional Table 1**

Summary of Theory of Planned Behaviour questionnaire items and internal reliability testing analysis plan

| **Construct** | **Number of questions** | **Coding** | **Reliability test** | **Item scoring** |
| --- | --- | --- | --- | --- |
| **Direct constructs** | | | | |
| Attitudes | 4 items | 1 to 7 | Cronbach’s alpha | Calculate mean score |
| Subjective norms | 3 items | 1 to 7 | Cronbach’s alpha | Calculate mean score |
| Perceived behavioural control | 3 items | 1 to 7 | Cronbach’s alpha | Calculate mean score |
| Generalised intention | 3 items | 1 to 7 | Cronbach’s alpha | Calculate mean score |
| Intention statement | 2 items | 0 to 10 |  | Calculate mean score |
