# Supplementary material for: Evaluating a handwashing with soap program in Australian remote Aboriginal communities: a pre and post intervention study design
Source: BMC Public Health. 2015 Nov 27;15:1188. doi: 10.1186/s12889-015-2503-x (PMC4662811; doi:10.1186/s12889-015-2503-x)
Supplement: Additional file 2: Table S2. — Reliability tests of constructs using Cronbach’s alpha statistical test*. (DOCX 14 kb) [file 12889_2015_2503_MOESM2_ESM.docx]

**Additional Table 2.**

Reliability tests of constructs using Cronbach’s alpha statistical test*

| **Construct** | **Number of respondents** | **Number of items in final scale** | **Alpha** |
| --- | --- | --- | --- |
| Attitudes | 766 | 4 | **.67** |
| Subjective norms | 766 | 3 | .40 |
| Perceived behavioural control | 766 | 3 | **.66** |
| Generalised intention | 766 | 3 | **.79** |

*Cronbach’s alpha coefficient value 0.6 or higher considered satisfactory
