# Supplementary material for: Evaluating a handwashing with soap program in Australian remote Aboriginal communities: a pre and post intervention study design
Source: BMC Public Health. 2015 Nov 27;15:1188. doi: 10.1186/s12889-015-2503-x (PMC4662811; doi:10.1186/s12889-015-2503-x)
Supplement: Additional file 3: Table S3. — Results of community level Paired Sample t-test (pre and post intervention) – Theory of Planned Behaviour constructs shown to have good internal reliability. (DOCX 20 kb) [file 12889_2015_2503_MOESM3_ESM.docx]

**Additional Table 3.**

Results of community level Paired Sample t-test (pre and post intervention) – Theory of Planned Behaviour constructs shown to have good internal reliability

|  | **Number of Participants** | **Mean*** | **Standard Deviation** | **Correlation** | **Significance** |
| --- | --- | --- | --- | --- | --- |
| **Attitude Overall** | | | | | |
| **Community 1** | | | | | |
| Pre | 14 | 6.14 | 1.58 | -0.229 | 0.430 |
| Post | 14 | 6.79 | 0.54 |  |  |
| **Community 2** | | | | | |
| Pre | 14 | 6.27 | 1.97 | -0.013 | 0.964 |
| Post | 14 | 6.30 | 0.58 |  |  |
| **Community 3** | | | | | |
| Pre | 31 | 6.48 | 1.31 | 0.467 | 0.008** |
| Post | 31 | 6.81 | 0.52 |  |  |
| **Community 4** | | | | | |
| Pre | 14 | 6.87 | 0.40 | -0.13 | 0.650 |
| Post | 14 | 6.48 | 1.03 |  |  |
| **Community 5** | | | | | |
| Pre | 21 | 6.51 | 0.99 | 0.753 | 0.000** |
| Post | 21 | 6.69 | 0.74 |  |  |
| **Community 6** | | | | | |
| Pre | 5 | 7.00 | 0.00 | -** | -*** |
| Post | 5 | 7.00 | 0.00 |  |  |
| **Perceived Behavioural Control** | | | | | |
| **Community 1** | | | | | |
| Pre | 14 | 5.45 | 1.60 | 0.309 | 0.283 |
| Post | 14 | 4.33 | 1.29 |  |  |
| **Community 2** | | | | | |
| Pre | 14 | 5.45 | 1.60 | -0.185 | 0.526 |
| Post | 14 | 4.33 | 1.29 |  |  |
| **Community 3** | | | | | |
| Pre | 31 | 6.48 | 0.84 | 0.175 | 0.346 |
| Post | 31 | 6.13 | 1.47 |  |  |
| **Community 4** | | | | | |
| Pre | 14 | 6.21 | 1.19 | 0.460 | 0.098 |
| Post | 14 | 5.36 | 2.54 |  |  |
| **Community 5** | | | | | |
| Pre | 21 | 5.40 | 1.96 | 0.371 | 0.097 |
| Post | 21 | 5.20 | 2.08 |  |  |
| **Community 6** | | | | | |
| Pre | 5 | 5.53 | 1.71 | -0.153 | 0.806 |
| Post | 5 | 6.60 | 0.89 |  |  |
| **Generalised Intention** | | | | | |
| **Community 1** | | | | | |
| Pre | 14 | 4.88 | 2.32 | -0.006 | 0.985 |
| Post | 14 | 5.57 | 2.28 |  |  |
| **Community 2** | | | | | |
| Pre | 14 | 5.42 | 2.24 | -0.38 | 0.177 |
| Post | 14 | 4.64 | 1.66 |  |  |
| **Community 3** | | | | | |
| Pre | 31 | 6.24 | 1.33 | 0.121 | 0.515 |
| Post | 31 | 6.50 | 1.02 |  |  |
| **Community 4** | | | | | |
| Pre | 14 | 6.40 | 0.98 | -0.441 | 0.115 |
| Post | 14 | 5.07 | 2.86 |  |  |
| **Community 5** | | | | | |
| Pre | 21 | 5.62 | 2.16 | 0.308 | 0.174 |
| Post | 21 | 6.05 | 1.83 |  |  |
| **Community 6** | | | | | |
| Pre | 5 | 6.60 | 0.89 | -0.242 | 0.695 |
| Post | 5 | 7.00 | 0.00 |  |  |

* Construct scale score range 1-7. **Significance at *p*=<0.05 level.***The correlation and *t* cannot be computed as the standard error of the difference is 0.
